# Geographic variation in malignant cardiac tumors and their outcomes: SEER database analysis

**DOI:** 10.3389/fonc.2023.1071770

**Published:** 2023-01-24

**Authors:** Mohamed Rahouma, Sherif Khairallah, Anas Dabsha, Massimo Baudo, Magdy M. El-Sayed Ahmed, Ivancarmine Gambardella, Christopher Lau, Yomna M. Esmail, Abdelrahman Mohamed, Leonard Girardi, Mario Gaudino, Roberto Lorusso, Stephanie L. Mick

**Affiliations:** ^1^ Cardiothoracic Surgery Departments, Weill Cornell Medicine, New York, NY, United States; ^2^ Surgical Oncology Department, National Cancer Institute, Cairo University, Cairo, Egypt; ^3^ Cardiac Surgery Department, Spedali Civili di Brescia, University of Brescia, Brescia, Italy; ^4^ Cardiothoracic Surgery Department, Mayo Clinic, Jacksonville, FL, United States; ^5^ Department of Surgery, Zagazig University Faculty of Medicine, Zagazig, Egypt; ^6^ Department of Cardio-Thoracic Surgery, Maastricht University Medical Centre, Maastricht University, Maastricht, Netherlands; ^7^ Cardiovascular Research Institute Maastricht (CARIM), Maastricht University, Maastricht, Netherlands

**Keywords:** primary malignant cardiac tumors, database analysis, geographic variation, cardiac surgery, oncology

## Abstract

**Introduction:**

Primary malignant cardiac tumors (PMCTs) are rare. Geographical distribution has been demonstrated to affect cancer outcomes, making the reduction of geographical inequalities a major priority for cancer control agencies. Geographic survival disparities have not been reported previously for PMCT and the aim of this study is to compare the prevalence and the long-term survival rate with respect to the geographic location of PMCTs using the Surveillance, Epidemiology, and End Results (SEER) research plus data 17 registries between 2000 and 2019.

**Methods:**

The SEER database was queried to identify geographic variation among PMCTs. We classified the included states into 4 geographical regions (Midwest, Northeast, South and West regions) based on the U.S. Census Bureau-designated regions and divisions. Different demographic and clinical variables were analyzed and compared between the four groups. Kaplan Meier curves and Cox regression were used for survival assessment.

**Results:**

A total of 563 patients were included in our analysis. The median age was 53 years (inter-quartile range (IQR): 38 - 68 years) and included 26, 90, 101, and 346 patients from the Midwest, Northeast, South, and West regions respectively. Sarcoma represented 65.6% of the cases, followed by hematological tumors (26.2%), while mesothelioma accounted for 2.1%. Treatment analysis showed no significant differences between different regions. Median overall survival was 11, 21, 13, and 11 months for Midwest, Northeast, South and West regions respectively and 5-year overall survival was 22.2%, 25.4%, 14.9%, and 17.6% respectively. On multivariate Cox regression, significant independent predictors of late overall mortality among the entire cohort included age (Hazard Ratio [HR] 1.028), year of diagnosis (HR 0.967), sarcoma (HR 3.36), surgery (HR 0.63) and chemotherapy (HR 0.56).

**Conclusion:**

Primary malignant cardiac tumors are rare and associated with poor prognosis. Sarcoma is the most common pathological type. Younger age, recent era diagnosis, surgical resection, and chemotherapy were the independent predictors of better survival. While univariate analysis revealed that patients in the South areas had a worse survival trend compared to other areas, geographic disparity in survival was nullified in multivariate analysis.

## Introduction

Primary malignant cardiac tumors (PMCTs) much less common than primary benign or secondary/metastatic cardiac tumors (1), although their existence dates back to the 15^th^ century (2). Most early reported cases have been postmortem diagnoses based on autopsy findings (3) due to a lack of advanced cardiac imaging and echocardiography in previous eras. The first antemortem diagnosis occurred in 1934 by Barnes et al (4) with the diagnosis made from electrocardiogram findings and biopsy of tumor embolic foci (4). In the recent era of advanced diagnostic imaging, an increase in the known incidence of cardiac tumors has been documented (5, 6). Sarcoma has been documented as the most common pathological type, followed by lymphoma and mesothelioma (5, 7, 8), with an increase in the incidence of cardiac lymphoma, especially in 1980s, and 1990s (Autoimmune deficiency Syndrome (AIDS) era) (1, 9), but this has been stable since 2000 due to marked improvement in the Human Immunodeficiency Virus (HIV) management (9). The incidence of cardiac mesothelioma has decreased due to less asbestos exposure (1, 10). The prognosis is generally poor, and complete surgical resection is the mainstay of the treatment, with better survival associated with complete resection in comparison to other treatment modalities (3). For instance, Simpson et al. reported a median survival of 17 months with resection versus 6 months with other modalities (3). The first successful surgery for PMCTs was reported by Maurer et al. in the year 1952 (11).

Area-level and geographic characteristics have been demonstrated to affect the cancer survival, independent of patient-level characteristics, making alleviation of the geographical inequalities in cancer outcomes a major priority for cancer control agencies (12). Understanding of the factors underlying geographical disparities is important for targeting policies. Geographic survival disparities have been reported previously in the setting of other malignant tumors (13–17), but not reported for PMCTs, therefore the aim of this study is to compare the geographic locations regarding the prevalence, and the long-term survival rate of PMCTs using the Surveillance, Epidemiology, and End Results (SEER) research plus registry 17.

## Material and methods

### Data sources

The SEER database of the National Cancer Institute (NCI) was queried to identify geographic variation among PMCTs patients based on inpatient status.

The public use version (https://www.cdc.gov/cancer/uscs/technical_notes/contributors/seer.htm) of data collected from the SEER research plus data 17 registries NOV-2021 (2000–2019) was used for this study. This registry is an extended form of the SEER data containing survival and treatment data and was released November of 2021 and includes patients from years 2000-2019. We used U.S. Census Bureau-designated regions and divisions as shown in **Supplementary Table 1** for the geographic classification of the included states in SEER database (18). The Census Bureau region definition is widely used for data collection and analysis and is the most used classification system. We included only patients with single primary cardiac tumor (sequence number = 0 or 1), as survival in patients with multiple primary tumors could not be ascribed to a single anatomical cancer site.

### Study population and inclusion criteria

Among our cohort, we classified the included states into 4 geographical regions based on the U.S. Census Bureau-designated regions and divisions as shown in **Supplementary Table 1** **(** 18). The Census Bureau region definition is widely used for data collection and analysis, and is the most commonly used classification system (18). There were 26 (4.6%) patients from the Midwest, 90 (16%) from Northeast, 101 (18%) from the South, and 346 (61.4%) from the West regions.

Patients were excluded if follow up data was missing or in case of presence of sequence of malignant neoplasms of more than 1 over the lifetime of the patient.

### Study variables

General baseline demographics and clinical variables included in the analysis are year of diagnosis, age, sex, race, histological subtype, stage, and treatment modalities (surgery, chemotherapy, or radiotherapy). Relevant socioeconomic variables were included in the analysis which included median income, area (nonmetropolitan or metropolitan), marital status, SEER registry state, and geographical region. Survival months and vital status, whether the patient was alive or dead at the last follow-up were retrieved.

### Survival and follow-up data

Overall survival (OS) was defined as the time from the date of diagnosis until the date of death from any cause or the date of the last follow-up. Overall median follow-up was 82 months (95%CI: 68-107). Median follow-up was 127 months in Midwest vs 64, 72 and 96 months in Northeast, South, and West regions respectively.

### Statistical analysis

Baseline demographics and clinical characteristics were compared between the four groups. Continuous variables are presented as median and interquartile range (IQR) and are compared between groups utilizing Kruskal-Wallis test. Categorical variables were presented as a frequency count and percentage and compared between groups using Chi-Squared test. Survival was estimated and presented using a Kaplan-Meier curve and compared across groups using the Log-Rank Test. Univariate and multivariate predictors of late mortality were estimated using the Cox proportional hazards method and are reported as a hazard ratio (HR) and 95% confidence interval (95%CI). Univariate predictors were selected for inclusion within the multivariate model if p<0.15.

All *p* values are two-sided and considered statistically significant if <0.05. The statistical analysis was performed using R version 4.2.1 within RStudio.

## Results

### Demographics

Among the 714 PMCTs cases in the SEER database, 27 cases were excluded due to missing survival time and 124 cases due to the presence of sequence of malignant neoplasms of more than 1 over the lifetime of the patient. Therefore, a total of 563 patients were included in the study analysis (**Supplementary Figure 1**).

Median age was 53 years (interquartile range (IQR): 38 to 68 years) and included 26, 90, 101 and 346 patients from Midwest, Northeast, South, and West regions respectively. Overall, there were 305 males (54.2%). More than three-quarters of the study population was White, followed by Asian race (11.2%), Black race (10.7%) and other (1.2%). Each stage (localized, regional, and distant) represented about one third of the included cohort (**Table 1**).

**Table 1:** Criteria of included studies.

	Overall	Midwest	Northeast	South	West	p
n	563	26	90	101	346	
**Age (median [IQR])**	53.00 [38.00, 68.00]	53.00 [37.00, 63.75]	56.00 [38.00, 75.00]	47.00 [35.00, 59.00]	54.00 [39.00, 68.00]	0.042
**Sex (Males (%))**	305 (54.2)	15 (57.7)	49 (54.4)	56 (55.4)	185 (53.5)	0.966
**Year of diagnosis (median [IQR])**	2011.00 [2006.00, 2015.00]	2013.00 [2005.00, 2016.75]	2012.00 [2006.00, 2016.75]	2011.00 [2007.00, 2015.00]	2010.00 [2006.00, 2015.00]	0.325
**Race (%)**						<0.001
**Asian or Pacific Islander**	63 (11.2)	1 (3.8)	11 (12.2)	5 (5.0)	46 (13.3)	0.072
**Black**	60 (10.7)	1 (3.8)	8 (8.9)	28 (27.7)	23 (6.6)	<0.001
**Others/Unknown**	7 (1.2)	0 (0.0)	0 (0.0)	0 (0.0)	7 (2.0)	0.217
**White**	433 (76.9)	24 (92.3)	71 (78.9)	68 (67.3)	270 (78.0)	0.027
**Histology (%)**						0.206
• Hematological tumor	150 (26.2)	4 (15.4)	34 (37.4)	22 (21.4)	90 (25.5)	0.034
• Mesothelioma	12 (2.1)	1 (3.8)	3 (3.3)	0 (0.0)	8 (2.3)	0.356
• Others/Unclassified	25 (4.4)	0 (0.0)	3 (3.3)	4 (3.9)	18 (5.1)	0.573
• Sarcoma	376 (65.6)	21 (80.8)	50 (54.9)	75 (72.8)	230 (65.2)	0.019
**Summary stage (%)**						0.163
• Distant	168 (34.4)	10 (47.6)	17 (23.9)	39 (42.4)	102 (33.6)	0.051
• Localized	155 (31.8)	5 (23.8)	29 (40.8)	25 (27.2)	96 (31.6)	0.2398
• Regional	134 (27.5)	6 (28.6)	17 (23.9)	23 (25.0)	88 (28.9)	0.784
• Unknown/unstaged	31 (6.4)	0 (0.0)	8 (11.3)	5 (5.4)	18 (5.9)	0.209
**SEER registry states (%)**						<0.001
• California	266 (47.2)	0 (0.0)	0 (0.0)	0 (0.0)	266 (76.9)	
• Connecticut	26 (4.6)	0 (0.0)	26 (28.9)	0 (0.0)	0 (0.0)	
• Georgia	56 (9.9)	0 (0.0)	0 (0.0)	56 (55.4)	0 (0.0)	
• Hawaii	10 (1.8)	0 (0.0)	0 (0.0)	0 (0.0)	10 (2.9)	
• Iowa	26 (4.6)	26 (100.0)	0 (0.0)	0 (0.0)	0 (0.0)	
• Kentucky	26 (4.6)	0 (0.0)	0 (0.0)	26 (25.7)	0 (0.0)	
• Louisiana	19 (3.4)	0 (0.0)	0 (0.0)	19 (18.8)	0 (0.0)	
• New Jersey	64 (11.4)	0 (0.0)	64 (71.1)	0 (0.0)	0 (0.0)	
• New Mexico	13 (2.3)	0 (0.0)	0 (0.0)	0 (0.0)	13 (3.8)	
• Seattle (Puget Sound)	41 (7.3)	0 (0.0)	0 (0.0)	0 (0.0)	41 (11.8)	
• Utah	16 (2.8)	0 (0.0)	0 (0.0)	0 (0.0)	16 (4.6)	
**Marital status (%)**						0.549
• Married	289 (51.3)	16 (61.5)	45 (50.0)	46 (45.5)	182 (52.6)	
• Not married	250 (44.4)	10 (38.5)	42 (46.7)	48 (47.5)	150 (43.4)	
• Unknown	24 (4.3)	0 (0.0)	3 (3.3)	7 (6.9)	14 (4.0)	
**Median income quartiles (%) <$50K**	48 (8.5)	2 (7.7)	0 (0.0)	33 (32.7)	13 (3.8)	<0.001
**Area (Non-metropolitan) (%)**	54 (9.6)	12 (46.2)	1 (1.1)	17 (16.8)	24 (6.9)	<0.001
**Surgery (%)**	292 (51.9)	13 (50.0)	43 (47.8)	45 (44.6)	191 (55.2)	0.227
**Radiotherapy (%)**	65 (11.5)	5 (19.2)	8 (8.9)	8 (7.9)	44 (12.7)	0.274
**chemotherapy (%)**	307 (54.5)	14 (53.8)	53 (58.9)	54 (53.5)	186 (53.8)	0.844
**Treatment bimodalities**						
• Surgery and Chemotherapy (%)	156 (27.7)	7 (26.9)	27 (30.0)	21 (20.8)	101 (29.2)	0.386
• Surgery and Radiotherapy (%)	62 (11.0)	5 (19.2)	8 (8.9)	7 (6.9)	42 (12.1)	0.224
• Chemotherapy and Radiotherapy(%)	65 (11.5)	5 (19.2)	8 (8.9)	8 (7.9)	44 (12.7)	0.274
• Treatment tri-modalities (Surgery and CRT) (%)	42 (7.5)	3 (11.5)	7 (7.8)	4 (4.0)	28 (8.1)	0.452
**Survival months (median [IQR])**	10.00 [2.00, 27.00]	10.00 [2.25, 29.25]	12.00 [3.00, 39.25]	10.00 [1.00, 22.00]	10.00 [2.00, 26.00]	0.537
**Vital status (Dead (%))**	440 (78.2)	22 (84.6)	62 (68.9)	80 (79.2)	276 (79.8)	0.124

¶Obtained using Chi-square test. P= 0.20 using Log rank test.

CRT, chemoradiation.

The four regions showed some significant differences: among the different regions, South region had younger patients with a median age of 47 vs 53 in Midwest (P=0.042). White race was the most prevalent and represented 92.3% vs 67.3% in Midwest vs South regions (P=0.027), while black race represented 27.7% vs 3.8% in South vs Midwest regions (P<0.001). One-third of South region has a median income of less than $50K vs zero percent in Northeast region (P<0.001). The South area had higher stages when compared to the Northeast area (P=0.051) (**Table 1**).

A subgroup analysis based on the number of treatment modalities showed that more treatments options were used in younger patients (p<0.001). Hematological tumors were more prevalent in the no treatment subgroup (p<0.001), while sarcoma was less prevalent in the no treatment subgroup (p<0.001). Patients receiving no treatment had a dismal prognosis with a median survival of 1 month (IQR: 0.0-5.0) (**Supplementary Table 2**).

The annual trend of PMCTs among the entire cohort, and the 4 regions is shown in **Supplementary Figure 2**.

### Histology and treatment

Among all patients, sarcoma was the most common histological category (65.6%), followed by hematological tumors (26.2%), while mesothelioma accounted for 2.1%. Sarcomas represented almost three-quarters of included cases in each of Midwest and South regions, while almost 60% in the other 2 regions (P=0.019). Hematological tumors were most common in Northeast region (37.4%, P=0.034) (**Table 1**).

A total of 292 patients (51.9%) underwent surgery, 307 (54.5%) received chemotherapy, and 65 (11.5%) received radiotherapy. Treatment analysis showed absence of significant differences between different regions either by studying each single modality, as bimodalities, or as tri-modalities (**Table 1**).

### Survival analysis

**Figure 1:**
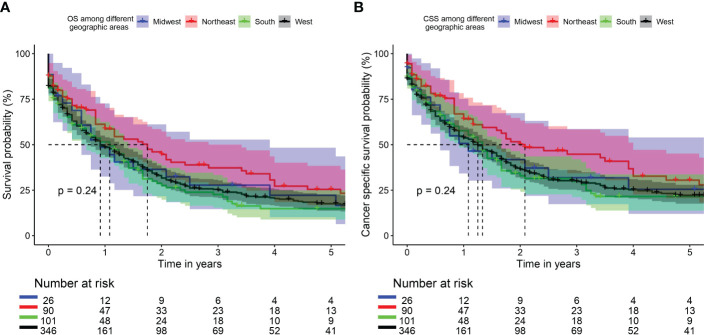
Kaplan Meier curve of overall survival (OS) **(A)** and cancer specific survival (CSS) **(B)** among different geographic areas.

Median overall survival was 11 months (95%CI: 7-47), 21 (95%CI: 12-41), 13 (95%CI:8-17), and 11 (95%CI: 9-16) for Midwest, Northeast, South, and West regions respectively. Two-year overall survival was 36.4%, 44.7%,27.6%, and 31.7% for Midwest, Northeast, South, and West regions respectively. 5-year overall survival was 22.2%, 25.4%, 14.9%, and 17.6% for Midwest, Northeast, South, and West regions respectively **(**Overall log rank P=0.24, **Figure 1A** **).**

Median cancer-specific survival was 13 months (95%CI: 7-47), 25 (95%CI: 16-48), 16 (95%CI:11-20), and 15 (95%CI: 11-19) for Midwest, Northeast, South, and West regions respectively. Two-year cancer-specific survival was 41.8%, 50.1%, 32.9%, and 36.4% for Midwest, Northeast, South, and West regions respectively. 5-year cancer-specific survival was 25.5%, 30.5%, 21.6%, and 22.2% for Midwest, Northeast, South, and West regions respectively **(**Overall log rank P=0.24, **Figure 1B** **).**

The median survival of surgery, chemotherapy and radiotherapy groups were 18 months (95%CI: 14-20), 20 months (95%CI: 16-22), and 23 months (95%CI: 16-38), respectively. Survival subgroup analysis on the number of treatments presented the following outcomes: “none” 1 month (95%CI: 0-2), “single modality” 13 months (95%CI: 9-16), “bimodality” 21 months (95%CI: 20-25), and “trimodality” 23 months (95%CI: 15-45).

On univariate Cox regression, South region was associated with a trend toward higher late mortality versus Northeast region (HR 1.39, 95%CI 0.99-1.93, P=0.054). On multivariable Cox regression, independent predictors of late overall mortality among the entire cohort included age (HR 1.028, 95%CI: 1.013-1.043, p<0.001), year of diagnosis (HR 0.967, 95%CI: 0.946-0.988, p=0.002), sarcoma (HR 3.36, 95%CI: 2.40;4.69, P<0.001, hematological tumor set as reference), surgery (HR 0.63, 95%CI: 0.48;0.81, p<0.001), and chemotherapy (HR 0.56, 95%CI 0.44-0.71, p<0.001) (**Table 2**).

**Table 2:** Predictors of late overall mortality among the included cohort using Cox regression.

	Univariate analysis¶	Multivariate analysis
Variable	HR (95CI) p-value	HR (95CI) p-value
**Age**	**1.006 [1.002;1.011] 0.008**	**1.028 [1.013;1.043] <0.001**
**Sex (Male vs Females)**	0.88 [0.73;1.06] 0.179	—–
**Year of diagnosis**	**0.980 [0.963;0.997] 0.024**	**0.967 [0.946;0.988] 0.002**
**Race (Ref: Black)**		
* **Asian or Pacific Islander**	0.76 [0.51;1.14] 0.184	0.774 [0.493;1.215] 0.266
* **Others/Unknown**	0.75 [0.27;2.07] 0.574	1.503 [0.523;4.319] 0.448
* **White**	0.81 [0.61;1.09] 0.165	0.748 [0.534;1.049] 0.092
**Histology (Ref: Hematological tumor)**		
* **Sarcoma**	**2.15 [1.69;2.73] <0.001**	**3.36 [2.40;4.69] <0.001**
**Summary stage (Ref: Distant)**		
* **Localized**	**0.57 [0.45;0.73] < 0.001**	0.54 [0.26;1.10] 0.091
* **Regional**	**0.69 [0.54;0.89] 0.004**	0.65 [0.27;1.52] 0.324
* **Unknown/unstaged**	1.27 [0.84;1.92] 0.247	3.73 [0.90;10.51] 0.120
**Median income quartiles (< vs ≥$50K)**	**1.38 [1.00;1.91] 0.051**	1.35 [0.90;2.04] 0.141
**Area (Nonmetropolitan vs Metropolitan)**	1.01 [0.74;1.38] 0.950	—–
**Marital status (Not married vs Married)**	1.03 [0.85;1.25] 0.757	—–
**Surgery (%)**	0.87 [0.72;1.05] 0.139	**0.63 [0.48;0.81] <0.001**
**Radiotherapy (%)**	**0.79 [0.59;1.06] 0.115**	0.94 [0.67;1.30] 0.718
**Chemotherapy (%)**	**0.51 [0.42;0.61] <0.001**	**0.56 [0.44;0.71] <0.001**
**Geographic areas (Ref: Northeast)**		
* **Midwest**	1.31 [0.80;2.13] 0.278	3.31 [0.35;30.6] 0.290
* **South**	**1.39 [0.99;1.93] 0.054**	1.63 [0.49;5.45] 0.423
* **West**	**1.29 [0.98;1.70] 0.071**	2.47 [0.89;6.85] 0.082
**Interaction terms**		
**Age : Geographic area (Midwest)**	—–	0.97 [0.93;1.02] 0.298
**Age : Geographic area (South)**	—–	0.99 [0.97;1.01] 0.582
**Age : Geographic area (West)**	—–	0.98 [0.97;1.00] 0.119
**Stage (Localized):Geographic area (Midwest)**	—–	0.66 [0.10;4.15] 0.664
**Stage (Regional):Geographic area (Midwest)**	—–	1.61 [0.39;6.50] 0.503
**Stage (Localized):Geographic area (South)**	—–	1.29 [0.51;3.24] 0.582
**Stage (Regional):Geographic area (South)**	—–	0.75 [0.26;2.12] 0.592
**Stage (Localized):Geographic area (West)**	—–	0.91 [0.42;1.99] 0.829
**Stage (Regional):Geographic area (West)**	—–	1.11 [0.44;2.75] 0.817

¶Variables with P ≤ 0.15 in univariate analysis were included in multivariate analysis.

In the “Univariate” column, the values in bold highlight the variables that were selected for multivariate analysis.

On multivariable Cox regression among surgical subgroup (n=292), independent predictors of late overall mortality included advanced age, earlier year of diagnosis, sarcoma vs hematological tumor set as reference, localized and regional stages vs distant stage, and chemotherapy (**Table 3**).

**Table 3:** Predictors of late overall mortality among the surgical subgroup using Cox regression.

	Multivariate analysis¶
Variable	HR (95CI) p-value
**Age**	1.015 [1.005;1.024] 0.002
**Year of diagnosis**	0.970 [0.942;0.999] 0.0433
**Race (Ref: Black)**	
• Asian or Pacific Islander	1.17 [0.61;2.24] 0.645
• Others/Unknown	2.07 [0.45;9.56] 0.351
• White	0.90 [0.54;1.51] 0.697
**Histology (Ref: Hematological tumor)**	
• Sarcoma	**4.43 [2.33;8.41] <0.001**
**Summary stage (Ref: Distant)**	
• Localized	**0.41 [0.28;0.59] <0.001**
• Regional	**0.56 [0.39;0.82] 0.003**
• Unknown/unstaged	0.76 [0.27;2.14] 0.599
**Median income quartiles (< vs ≥$50K)**	0.95 [0.53;1.68] 0.855
**Area (Nonmetropolitan vs Metropolitan)**	—–
**Marital status (Not married vs Married)**	—–
**Radiotherapy (%)**	0.91 [0.65;1.27] 0.581
**Chemotherapy (%)**	**0.72 [0.53;0.99] 0.040**
**Geographic areas (Ref: Northeast)**	
• Midwest	0.65 [0.29;1.45] 0.293
• South	1.52 [0.84;2.76] 0.165
• West	1.32 [0.84;2.07] 0.224

¶Variables with P ≤ 0.15 in univariate analysis were included in multivariate analysis.

In the “Multivariate” column, the values in bold highlight the significant variables.

A graphical summary of the findings of our study is represented in **Supplementary Figure 3**.

## Discussion

Primary malignant cardiac tumors are rare and only account for 0.008% of reported cancer, and 9.4% of all primary cardiac tumors in the SEER database (1, 19). While prior series reported that secondary cardiac tumors (SCTs) are 20 to 30 times more common than primary cardiac tumors, with the most common tumor source being hematological malignancies (leukemia and lymphoma), and other solid tumors especially melanoma, lung, and breast cancer (6), recent meta-analysis reported almost equal prevalence of both primary and SCTs (19). A low incidence and prevalence of PMCTs has been demonstrated over different decades (5, 20–27). In the recent era, despite their rarity, the incidence of these tumors appeared to have increased due to recent advance in the cardiac imaging (MRI and CT) (1). Moreover, cardiac imaging can evaluate the characteristics of cardiac tumors by visualizing the relationship between the tumor and the surrounding tissues, and is essential for the surgical plan, the assessment of tumor progression, and the monitoring of postoperative tumor recurrence and metastasis (28). Angiosarcoma was and still the most common, followed by lymphoma (especially in immunocompromised patients; AIDS, and following organ transplant) (5, 7, 8).

Due to the relative scarcity of reported cases of these tumors, there is no conclusive study showing any survival disparities between geographical locations in these patients. Therefore, the objective of this study was to assess survival outcomes for different geographical locations in United States based on the SEER data.

In univariate analysis, a worse survival trend for patients in the South area compared to patients in the Northeast area (HR: 1.39, 95%CI 0.99-1.93], p = 0.054) was observed. However, at multivariable analysis, none of the geographic areas showed a significant disparity in long term survival. With respect to the demographic and the clinicopathological characteristics between these 4 geographic locations, we found that patients in South region (worst survival in univariate analysis) were relatively younger in age, with relatively lower income (p<0.001) and higher tumor stages (p=0.051) when compared to the Northeast region (the best survival). There were more patients from non-metropolitan areas (p<0.001) compared to Northeast area. There was no difference in sex distribution or treatment options provided between the groups. In the whole cohort, the most common racial designation was Caucasian. The South region had a higher proportion of black patients as compared to Northeast. There is no evidence that racial difference affects the long-term survival among patients with cardiac tumors (29, 30). In previous reports about impact of geographic location on cancer survival, the authors surmised that the survival advantages of one geographic region area over others was mainly attributed to socioeconomic status, income and education levels, healthcare facilities inequalities, and less access to medical care (in rural vs. urban areas) rather than racial differences (12–17). While prior series showed better survival for patients with private insurance/managed care insurance vs Medicare (HR= 0.67 (95%CI 0.52-0.87), P=0.002) (31), we were not able to investigated these factors as the SEER version used did not contain data on insurance, education levels, or healthcare facility type. In private insurance countries, the economic disparity may impact the outcomes of the patients as non-insured patients are less likely to receive costly treatments. However, also countries with national healthcare system may not always guarantee a full equity of care (32). The health insurance status is also related to the socioeconomic status. In fact, in clinical trials where treatments are free, low socioeconomic status is associated with worse outcomes (33). Besides, nationwide cardiovascular studies have reported that patients with a low socioeconomic status may be under- or late diagnosed and have limited access to several treatments (34, 35).

In PMCT, age has proven to negatively impact survival outcomes of a prior analysis of the National Cancer Database (36).With increasing age, patients presented a more significant comorbidity burden compared to younger ones and were treated more conservatively. This was confirmed also in the current analysis were patients that did not receive any treatment were significantly older than the other treatment subgroups (p<0.001).

Sarcoma was the most common pathological type (65.6%), followed by hematological malignancies (26.2%) and mesothelioma (2.1%) in the whole cohort. There was a higher percent of sarcoma in the Southern region. Our results from the SEER database are similar to reports from non-American databases regarding the incidence of tumor types of PMCT (5, 20–22, 26). Blondeau et al. in a French study of 533 cases reported that sarcoma represented the majority of PMCT (90%) (20). Approximately 50% of our cohort underwent surgery, 53% received chemotherapy, and 11.3% of patients underwent radiation therapy. The reasons for non-cancer directed surgery was mentioned in **Supplementary Table 3**. Surgery has been established to be the most effective treatment option for PMCTs (3), which can also be performed through minimally invasive approaches in selected patients. Sultan and colleagues in their multi-institutional study from the National Cancer Database confirm that the surgery group as compared to the no surgery groups had significantly better long-term survival (p<0.0001) (37). Simpson et al. reported a median survival of 17 months for those who underwent surgery compared with only 6 months in patients received other treatment options (3). Chemotherapy was used for advanced non operable cases, or as a neoadjuvant before surgery, and the reasons why radiotherapy was not widely used might be related to radio-insensitivity of some cardiac sarcomas, as well as its cardiac-related-toxicity which furthermore deteriorate the cardiac functions (38). However, previous studies have shown that radiotherapy was associated with improved progression-free survival (PFS) on multivariate analysis (39).

Randhawa et al. reported their 25 years’ experience that patients who received multimodality treatment (any combination of surgery, radiation therapy, and chemotherapy) had an improved median survival compared with patients treated with surgery, radiation therapy, or chemotherapy only (P=0.05) (40). This was also reported in other studies (41, 42). Saleh and colleagues showed that neoadjuvant chemotherapy followed by radical surgery is safe and effective strategy also in patients with right-sided heart sarcomas (43). Besides, due to the propensity for brain metastases in cardiac tumors, brain MRI at the time of diagnosis should be considered (44).

Primary malignant cardiac tumors are generally associated with poor outcomes, and 78.2% of patients in our analysis were dead at the end of follow-up, with median survival of 10 months. In multivariate analysis, older age, earlier era diagnosis, sarcoma, and non-surgical treatment options were associated with poor survival. Our results are similar to those reported by Bui et al (29) and Yin et al (30). Bui et al (29) demonstrated survival improvement in patients diagnosed in the recent era compared to old era (The 1-year survival rate: 13.3% (1975–1998), 40.9% (1999–2004), 50% (2005–2010), and 59.7% (2011–2016), *p*-value = 0.0064), however, Yin et al (30) study did not show this survival advantages although there was an obvious trend (*P* = 0.13).

### Limitations

This study utilized the SEER database, which is considered a highly reliable source of epidemiologic information (incidence and prevalence), and survival assessment. However, inaccurate estimation of the prevalence of cardiac tumors in the SEER data might be present as these tumors can present by sudden cardiac death (45), hence not documented, as the SEER data reported only living cases not the postmortem diagnosis. Studying the impact of the geographic location on survival from the SEER data might be changed over time given the fact of patients’ migration to different areas. Assessment of comorbidity information, genetic details or individual risk factors were not reported in the SEER data. We used a SEER version that had both states and survival data but no insurance, education levels, or healthcare facilities type data. Furthermore, the details of the received treatment such as the doses, local and systemic side effects of the chemotherapy or radiotherapy were not reported, so its impact on survival cannot be assessed.

### Conclusion

Primary malignant cardiac tumors are rare and associated with poor prognosis. Sarcoma is the most common pathological type. Younger age, recent era diagnosis, surgical resection, and chemotherapy are identified as the independent predictors of better survival. While univariate analysis suggested that patients in the Southern region had worse survival trend compared to other geographic areas, the geographic disparity in survival was nullified in multivariate analysis.

## Data availability statement

The original contributions presented in the study are included in the article/**Supplementary Material**. Further inquiries can be directed to the corresponding author.

## Ethics statement

Ethical review and approval was not required for the study on human participants in accordance with the local legislation and institutional requirements. Written informed consent for participation was not required for this study in accordance with the national legislation and the institutional requirements.

## Author contributions

Conceptualization: MR, SK, MB. Methodology: MR. Software, Validation, Formal analysis: MR. Data curation: MR, SK, AD, MB, MA, IG, CL, YME. Investigation: MR, SK, MB. Writing-review and editing: All authors. Visualization: MR, SK, AD, MB, MA, IG, CL, YME. Resources: MR, MA, SM. Supervision: MR, SK, MA, CL, RL, SM. Project administration: MR, SK, MA, SM.

## Supplementary material

Supplementary Table 1 U.S. Census Bureau-designated regions and divisions.

Supplementary Table 2 Criteria of included studies based on the number of treatments received.

Supplementary Table 3 Reason for no cancer directed surgery.

Supplementary Figure 1 Flowchart of our included cases.

Supplementary Figure 2 Annual trend of PMCTs among **(A)** the entire cohort, **(B)** Midwest, **(C)** Northeast, **(D)** South, and **(E)** West regions.

Supplementary Figure 3 Graphical summary of the geographical findings.
