# Supplementary material for: Geographic variation in malignant cardiac tumors and their outcomes: SEER database analysis
Source: Front Oncol. 2023 Jan 24;13:1071770. doi: 10.3389/fonc.2023.1071770 (PMC9902931; doi:10.3389/fonc.2023.1071770)
Supplement: Supplementary Table 1 — U.S. Census Bureau-designated regions and divisions. [file DataSheet_1.zip › Supplementary Tables.docx]

**Supplementary Table 1: U.S. Census Bureau-designated regions and divisions (34)**

| - Region 1: Northeast   - Division 1: New England (Connecticut, Maine, Massachusetts, New Hampshire, Rhode Island, and Vermont)   - Division 2: Mid-Atlantic (New Jersey, New York, and Pennsylvania) - Region 2: Midwest   - Division 3: East North Central (Illinois, Indiana, Michigan, Ohio, and Wisconsin)   - Division 4: West North Central (Iowa, Kansas, Minnesota, Missouri, Nebraska, North Dakota, and South Dakota) - Region 3: South   - Division 5: South Atlantic (Delaware; Florida; Georgia; Maryland; North Carolina; South Carolina; Virginia; Washington, D.C. and West Virginia)   - Division 6: East South Central (Alabama, Kentucky, Mississippi, and Tennessee)   - Division 7: West South Central (Arkansas, Louisiana, Oklahoma, and Texas) - Region 4: West   - Division 8: Mountain (Arizona, Colorado, Idaho, Montana, Nevada, New Mexico, Utah, and Wyoming)   - Division 9: Pacific (Alaska, California, Hawaii, Oregon, and Washington) |
| --- |

**Supplementary Table 2: Criteria of included studies based on the number of treatments received**

|  | None | Single modality | Bimodality | Trimodality | P value |
| --- | --- | --- | --- | --- | --- |
| n | 120 | 264 | 137 | 42 |  |
| Age (median [IQR]) | 64.00 [49.00, 81.25] | 54.00 [38.00, 67.00] | 46.00 [32.00, 60.00] | 48.50 [37.00, 54.75] | <0.001 |
| Sex (Males (%)) | 73 (60.8) | 136 (51.5) | 77 (56.2) | 19 (45.2) | 0.215 |
| Year of diagnosis (median [IQR]) | 2011.00 [2007.00, 2016.00] | 2010.00 [2005.00, 2014.25] | 2012.00 [2006.00, 2016.00] | 2012.00 [2007.25, 2014.00] | 0.037 |
| Race (%) |  |  |  |  | 0.571 |
| - Asian or Pacific Islander | 12 (10.0) | 26 (9.8) | 17 (12.4) | 8 (19.0) | 0.326 |
| - Black | 11 (9.2) | 33 (12.5) | 14 (10.2) | 2 (4.8) | 0.426 |
| - Others/Unknown | 1 (0.8) | 5 (1.9) | 1 (0.7) | 0 (0.0) | 0.594 |
| - White | 96 (80.0) | 200 (75.8) | 105 (76.6) | 32 (76.2) | 0.835 |
| Histology (%) |  |  |  |  | <0.001 |
| - Hematological tumor | 40 (33.3) | 89 (33.7) | 17 (12.4) | 4 (9.5) | <0.001 |
| - Mesothelioma | 5 (4.2) | 4 (1.5) | 3 (2.2) | 0 (0.0) | 0.286 |
| - Others/Unclassified | 18 (15.0) | 5 (1.9) | 2 (1.5) | 0 (0.0) | <0.001 |
| - Sarcoma | 57 (47.5) | 166 (62.9) | 115 (83.9) | 38 (90.5) | <0.001 |
| Summary stage (%) |  |  |  |  | <0.001 |
| - Distant | 36 (36.4) | 78 (32.5) | 40 (35.7) | 14 (37.8) | 0.842 |
| - Localized | 23 (23.2) | 92 (38.3) | 30 (26.8) | 10 (27.0) | 0.021 |
| - Regional | 20 (20.2) | 61 (25.4) | 40 (35.7) | 13 (35.1) | 0.045 |
| - Unknown/unstaged | 20 (20.2) | 9 (3.8) | 2 (1.8) | 0 (0.0) | <0.001 |
| SEER registry states (%) |  |  |  |  | 0.437 |
| - California | 51 (42.5) | 120 (45.5) | 73 (53.3) | 22 (52.4) |  |
| - Connecticut | 8 (6.7) | 11 (4.2) | 4 (2.9) | 3 (7.1) |  |
| - Georgia | 13 (10.8) | 30 (11.4) | 11 (8.0) | 2 (4.8) |  |
| - Hawaii | 5 (4.2) | 3 (1.1) | 0 (0.0) | 2 (4.8) |  |
| - Iowa | 6 (5.0) | 11 (4.2) | 6 (4.4) | 3 (7.1) |  |
| - Kentucky | 6 (5.0) | 13 (4.9) | 5 (3.6) | 2 (4.8) |  |
| - Louisiana | 4 (3.3) | 10 (3.8) | 5 (3.6) | 0 (0.0) |  |
| - New Jersey | 13 (10.8) | 30 (11.4) | 17 (12.4) | 4 (9.5) |  |
| - New Mexico | 5 (4.2) | 6 (2.3) | 1 (0.7) | 1 (2.4) |  |
| - Seattle (Puget Sound) | 5 (4.2) | 25 (9.5) | 8 (5.8) | 3 (7.1) |  |
| - Utah | 4 (3.3) | 5 (1.9) | 7 (5.1) | 0 (0.0) |  |
| Marital status (%) |  |  |  |  | 0.310 |
| - Married | 58 (48.3) | 132 (50.0) | 71 (51.8) | 28 (66.7) |  |
| - Not married | 54 (45.0) | 123 (46.6) | 61 (44.5) | 12 (28.6) |  |
| - Unknown | 8 (6.7) | 9 (3.4) | 5 (3.6) | 2 (4.8) |  |
| Median income quartiles (%) <$50K | 11 (9.2) | 21 (8.0) | 13 (9.5) | 3 (7.1) | 0.932 |
| Area (Non-metropolitan) (%) | 8 (6.7) | 26 (9.8) | 13 (9.5) | 7 (16.7) | 0.304 |
| Surgery (%) | 0 (0.0) | 116 (43.9) | 134 (97.8) | 42 (100.0) | <0.001 |
| Radiotherapy (%) | 0 (0.0) | 0 (0.0) | 23 (16.8) | 42 (100.0) | <0.001 |
| Chemotherapy (%) | 0 (0.0) | 148 (56.1) | 117 (85.4) | 42 (100.0) | <0.001 |
| Treatment bimodalities |  |  |  |  |  |
| - Surgery and Chemotherapy (%) | - | - | 114 (83.2) | - |  |
| - Surgery and Radiotherapy (%) | - | - | 20 (14.6) | - |  |
| - Chemotherapy and Radiotherapy (%) | - | - | 3 (2.2) | - |  |
| Survival months (median [IQR]) | 1.00 [0.00, 5.00] | 10.50 [2.00, 35.25] | 19.00 [10.00, 29.00] | 16.00 [9.50, 30.75] | <0.001 |
| Vital status (Dead (%)) | 100 (83.3) | 207 (78.4) | 102 (74.5) | 31 (73.8) | 0.326 |

**Supplementary Table 3: Reason for no cancer directed surgery**

|  | Overall | Midwest | Northeast | South | West |
| --- | --- | --- | --- | --- | --- |
| n | 563 | 26 | 90 | 101 | 346 |
| Not performed, patient died prior to recommended surgery | 3 (0.5) | 1 (3.8) | 1 (1.1) | 1 (1.0) | 0 (0.0) |
| Not recommended | 243 (43.2) | 10 (38.5) | 40 (44.4) | 52 (51.5) | 141 (40.8) |
| Not recommended, contraindicated due to other conditions | 9 (1.6) | 1 (3.8) | 3 (3.3) | 1 (1.0) | 4 (1.2) |
| Recommended but not performed, patient refused | 2 (0.4) | 0 (0.0) | 0 (0.0) | 1 (1.0) | 1 (0.3) |
| Recommended but not performed, unknown reason | 12 (2.2) | 0 (0.0) | 3 (3.3) | 0 (0.0) | 9 (2.6) |
| Recommended, unknown if performed | 2 (0.4) | 1 (3.8) | 0 (0.0) | 1 (1.0) | 0 (0.0) |
| Surgery performed | 292 (51.9) | 13 (50.0) | 43 (47.8) | 45 (44.6) | 191 (55.2) |
