# Supplementary figures and images for: Geographic variation in malignant cardiac tumors and their outcomes: SEER database analysis
Source: Front Oncol. 2023 Jan 24;13:1071770. doi: 10.3389/fonc.2023.1071770 (PMC9902931; doi:10.3389/fonc.2023.1071770)

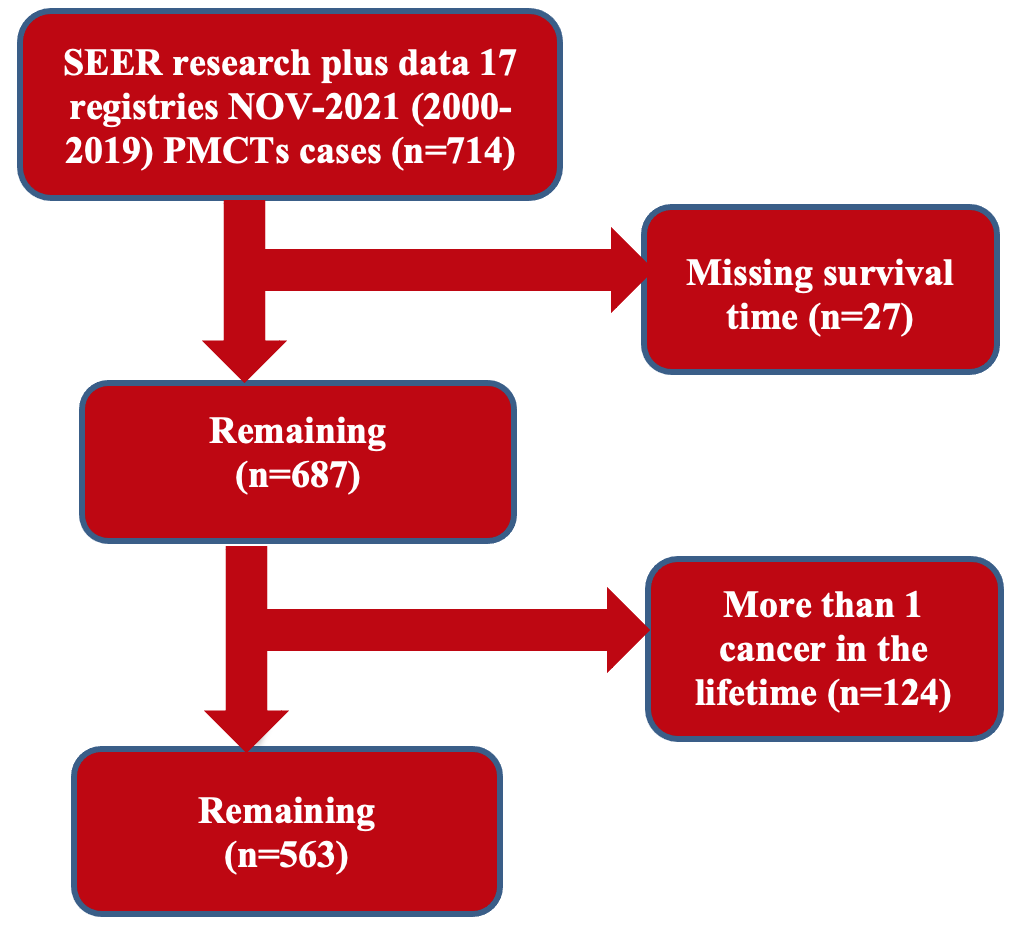

Supplement: Supplementary Table 1 — U.S. Census Bureau-designated regions and divisions. [file DataSheet_1.zip › Supplementary Figure 1.tiff]

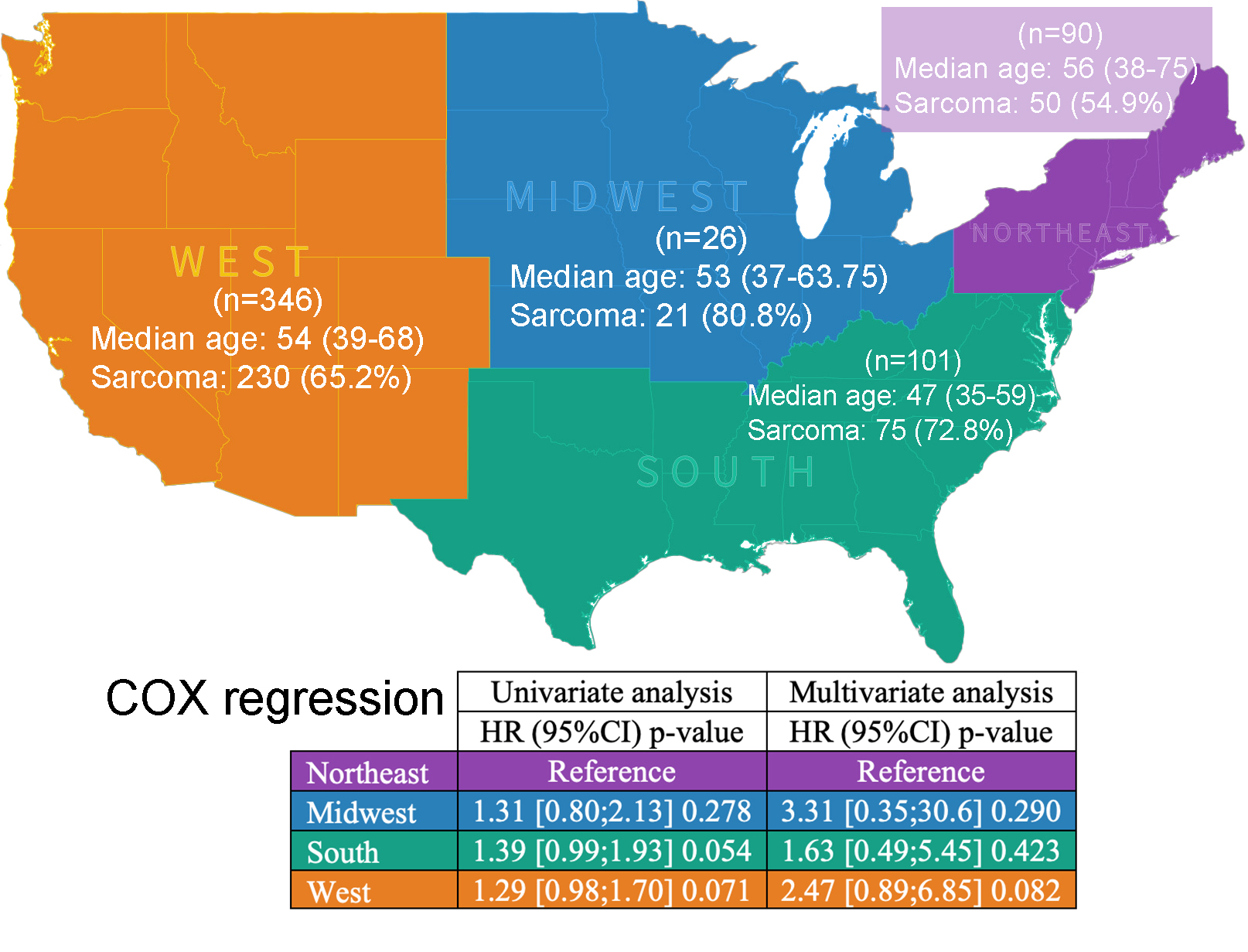

Supplement: Supplementary Table 1 — U.S. Census Bureau-designated regions and divisions. [file DataSheet_1.zip › Supplementary Figure 3.jpg]

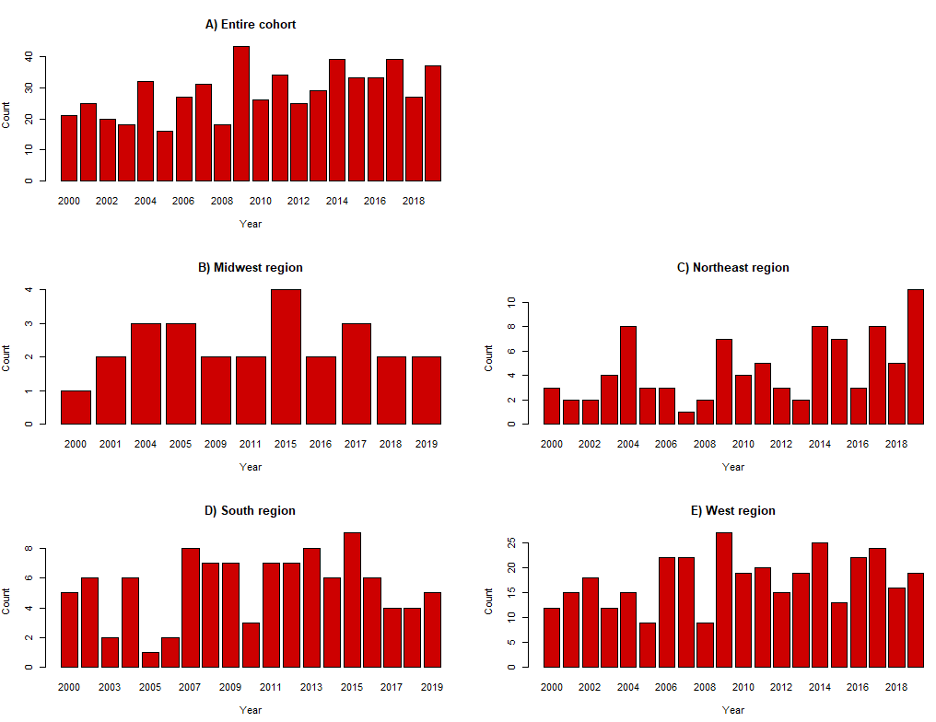

Supplement: Supplementary Table 1 — U.S. Census Bureau-designated regions and divisions. [file DataSheet_1.zip › Supplementary Figure 2.jpg]
